# Shifting paradigms: a pivotal study on laparoscopic resection for colovesical fistulas in diverticular disease

**DOI:** 10.3389/fsurg.2024.1370370

**Published:** 2024-03-01

**Authors:** Antonia Rizzuto, Jacopo Andreuccetti, Umberto Bracale, Vania Silvestri, Emanuele Pontecorvi, Stefano Reggio, Carlo Sagnelli, Roberto Peltrini, Angela Amaddeo, Cristina Bozzarello, Giusto Pignata, Diego Cuccurullo, Francesco Corcione

**Affiliations:** ^1^ Department of Medical and Surgical Science, University of Magna Graecia, Catanzaro, Italy; ^2^ Department of General Surgery, Civil Hospital of Brescia, Brescia, Italy; ^3^ Department of Medicine, University of Salerno, Fisciano, Italy; ^4^ Department of Public Health, School of Medicine and Surgery, University of Naples Federico II, Naples, Italy; ^5^ Department of General, Laparoscopic and Robotic Surgery, Monaldi Hospital, Naples, Italy

**Keywords:** diverticular disease, colovesical fistula, enterovesical fistula, laparoscopic sigmoidectomy, minimally invasive surgery, colorectal surgery

## Abstract

**Background:**

Colovesical fistulas (CVFs) pose a challenge in diverticulitis, affecting 4% to 20% of sigmoid colon cases. Complicated diverticular disease contributes significantly, accounting for 60%−70% of all CVFs. Existing studies on laparoscopic CVF management lack clarity on its effectiveness in diverticular cases compared to open surgery. This study redefines paradigms by assessing the potentiality, adequacy, and utility of laparoscopy in treating CVFs due to complicated diverticular disease, marking a paradigm shift in surgical approaches.

**Methods:**

Conducting a retrospective analysis at Ospedale Monaldi A.O.R.N dei Colli and University Federico II, Naples, Italy, patients undergoing surgery for CVF secondary to diverticular disease between 2010 and 2020 were examined. Comprehensive data, including demographics, clinical parameters, preoperative diagnoses, operative and postoperative details, and histopathological examination, were meticulously recorded. Patients were classified into open surgery (Group A) and laparoscopy (Group B). Statistical analysis used IBM SPSS Statistic 19.0.

**Results:**

From January 2010 to December 2020, 76 patients underwent surgery for colovesical fistula secondary to diverticular disease. Laparoscopic surgery (Group B, *n* = 40) and open surgery (Group A, *n* = 36) showed no statistically significant differences in operative time, bladder suture, or associated procedures. Laparoscopy demonstrated advantages, including lower intraoperative blood loss, reduced postoperative primary ileus, and a significantly shorter length of stay. Postoperative morbidity differed significantly between groups. Mortality occurred in Group A but was unrelated to surgical complications. No reoperations were observed. Two-year follow-up revealed no fistula recurrence.

**Conclusion:**

This pivotal study marks a paradigm shift by emphasizing laparoscopic resection and primary anastomosis as a safe and feasible option for managing CVF secondary to diverticular disease. Comparable conversion, morbidity, and mortality rates to the open approach underscore the transformative potential of these findings. The study's emphasis on patient selection and surgeon experience challenges existing paradigms, offering a progressive shift toward minimally invasive solutions.

## Introduction

Diverticular disease, particularly affecting the sigmoid colon, poses a challenge for 10%–25% of individuals with this condition (1, 2). Complications arise in about 15% of diverticular disease cases (3–5). Among the management options for these complications, laparoscopic colectomy has become the leading choice globally for addressing symptomatic sigmoid diverticulitis (6–9).

**Figure 1:**
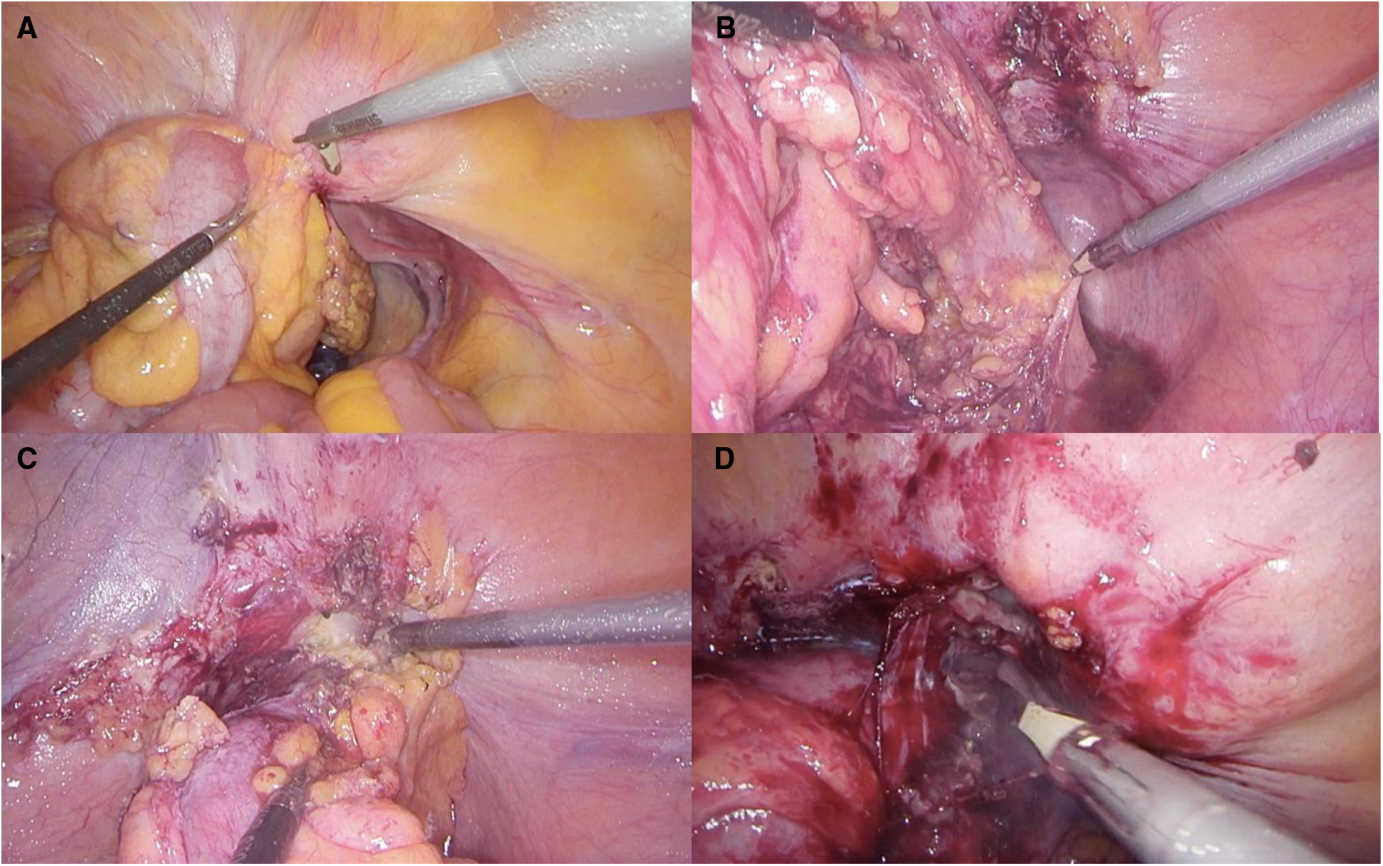
(**A**) Colovesical fistula visualization; (**B**) preparation of the posterior section of the colon (**C**) preparation of the anterior section of the colon (**D**)dissection of fistulous communication.

One significant complication associated with diverticular disease is the occurrence of colovesical fistulas (CVFs) (Figure 1) happening in 4%–20% of cases and representing a substantial majority, around 60%–70%, of all CVF instances (10, 11). The causes of these fistulas are complex, involving the direct extension of a perforated diverticulum or erosion through the bladder wall (12, 13). This complexity is further influenced by the anatomical intricacies of the pelvis, physiological functions, and the distinct pressure gradient between the bowel and the bladder (14).

Clinical symptoms often manifest as lower urinary tract issues, with patients reporting problems such as pneumaturia, fecaluria, and recurrent urinary tract infections. These symptoms highlight the intricate pathological mechanism involved in CVF development (15). Traditionally, CVFs were considered a historical contraindication to minimally invasive approaches due to concerns about safety and feasibility (10). However, recent reports, such as those by Badic et al. (11), challenge this belief, emphasizing the safety and feasibility of laparoscopic management, even though there are higher conversion rates and associated morbidity.

Despite the growing literature on laparoscopic management of CVFs, there is still a significant gap in our understanding of the actual effectiveness and utility of the minimally invasive approach compared to open surgery (16). Adding to this challenge, recent studies may lack definitive conclusions as they often include a diverse group of patients with CVFs of varying causes, failing to distinguish between those secondary to diverticular disease and those arising from different factors (16).

This report aims to address these knowledge gaps by focusing on evaluating the adequacy and utility of laparoscopy in treating CVFs complicating diverticular disease, directly comparing it to open surgery. By examining this specific group of patients, our goal is to provide nuanced insights that can guide clinicians and surgeons in optimizing their approach to these intricate cases.

## Material and methods

### Patient recruitment

A retrospective analysis included 410 patients who underwent surgery for diverticular disease from 2010 to 2020 at Ospedale Monaldi A.O.R.N dei Colli and University Federico II, Naples, Italy. Demographic and clinical data were collected from CPT codes. Eligible participants were 18 years or older without contraindications for major elective surgery. Patients with colovesical fistula (CVF) were divided into two groups: those undergoing open surgery (Group A) and laparoscopy (Group B).

### Data collection

Prospective recording covered demographic details, clinical parameters, preoperative diagnoses, operative data (operative time, procedure specifics, anastomosis type, conversion rates, intraoperative complications), postoperative outcomes (complications graded by Clavien-Dindo classification, postoperative ileus, hospital stay, reintervention, mortality), and histopathological examination. Routine preoperative examinations included blood tests, cardiological examination, chest x-rays, CT scans, colonoscopy, cystoscopy, and abdominal ultrasounds. Specific informed consent was obtained from each patient.

### Preoperative and postoperative management

No mechanical bowel preparation was administered, and no preoperative diet restrictions were applied. Deep venous thrombosis (DVT) prophylaxis included early mobilization and Low Molecular Weight Heparin (LMWH). Antimicrobials were given within 1 h before incision. Postoperatively, patients were allowed to drink on the first day if tolerated, and oral nutritional support was initiated from the second day onwards. Antiemetics were administered regularly for 72 h postoperatively. Discharge criteria included the return of bowel function, absence of nausea or vomiting, tolerance of oral intake, no abdominal distention, absence of complications, adequate mobility, and patient acceptance.

### Laparoscopic surgical technique

General anesthesia was administered, and patients were placed supine with abducted legs in a mild reverse Trendelenburg position. The procedure was conducted with a totally laparoscopic approach. Pneumoperitoneum was established using the open Veress-assisted technique with a 30-degree scope. Dissection utilized atraumatic graspers and an ultrasonic energy device. The surgical steps of laparoscopic sigmoid colectomy, including splenic flexure takedown and colonic mobilization, were performed. The Inferior Mesenteric Artery (IMA) was divided after exposing the common arterial trunk and its branches. The mesenteric defect was closed using fibrin glue. No drain was placed as per the standard approach in colorectal surgery. The specimen was extracted through an enlargement of the suprapubic port site. When technically feasible, Sigmoid Colectomy with IMA Preservation for Diverticular Disease was performed. Bladder wall repair was conducted for patients with a positive leak test.

### Statistical analysis

The Mann–Whitney *U*-test and Fisher exact tests were employed for statistical analysis, considering a *P*-value < 0.05 as significant. IBM SPSS Statistics 19.0 software facilitated data analysis.

## Results

Between 2010 and 2020, 76 patients (29 males, 46 females) underwent surgery for colovesical fistula secondary to complicated diverticulitis. Of these, 40 underwent laparoscopic surgery (Group B), and 36 had open surgery (Group A). Most patients presented with pathognomonic signs of CVF.

Refer to Table 1 for an overview of demographic data. Comprehensive details on patient demographics are provided in Table 2.

**Table 1:** Presenting complaints of patients undergoing surgery for colovesical fistulas secondary to diverticular disease.

Presenting complaint	Patients (%)	
Recurrent urinary tract infections.	54	71.0
Pneumaturia	34	44.7
Abdominal pain	16	21
Fecaluria	25	32.8
Diarrhea	8	10.5
Septicemia	8	10.5

**Table 2:** Demographic data and outcome of patients undergoing surgery for colovesical fistula secondary to diverticular disease.

	Open surgery (*n* = 36)	Laparoscopic surgery (*n* = 40)	*P*
Age (year)	Mean 69.21 (52–88)	Mean 65.45 (46–88)	0.1687 n.s.
Sex (M/F)	14/22	15/25	0.63 ns
ASA	30/36	16/43	0.45 ns
Previous abdominal Surgery %	18/36 50%	26/40 65%	0.94 ns.
Body mass index	25.32 ± 0.75	25.50 ± 0.65	0.858 ns.
Operative TIME (m)	164.8 ± 11.22 (75/300)	173.5 ± 9.8 (95/350)	0.56 ns
Bladder suture %	31/33 93.9%	32/40 80%	0.168 ns.
Urinary catether (d)	13.35 ± 1.12	11.77 ± 1.12	0.33 ns
Dindo clavien (1–5)	2.68 ± 0.21	1.56 ± 0.14	<0.0001
Blood loss (ml)	115.9 ± 20.35	73.21 ± 5.08	0.04
Prolonged ileus (d)	2.67 ± 0.13	2.23 ± 0.12	0.003
Associated surgical Procedures (pt)	11 (30.5.9%)	23 (57.5%)	0.09
Total hospital Stay (d)	10.79 ± 0.88	7.24 ± 0.36	<0.0001

### Preoperative findings

**Figure 2:**
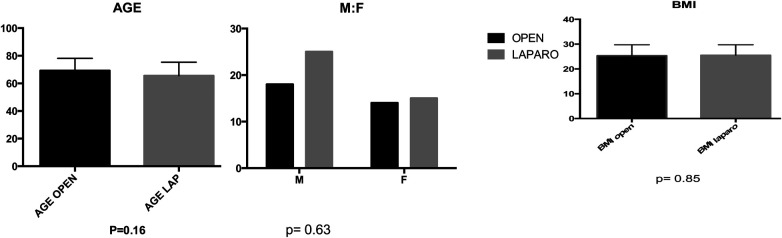
In the comparative analysis of two groups, comprising age, sex, and BMI, no statistically significant differences were observed, with p-values exceeding 0.05 for (**A**)age, (**B**) sex distribution, and (**C**) body mass index (BMI). A (*p* = 0.16) B (*p* = 0.63) C (*p* = 0.85).

Groups A and B demonstrated similar preoperative demographics, including median age (69 vs. 65 years), sex distribution (M:F 14/22 vs. 15/25), and BMI (25.33 vs. 25.50) (Figure 2). Concomitant colovaginal fistula occurred in 9.2%, and 3.9% had a concomitant ileovesical fistula. More than half of the patients in both group A and B (*P* = 0.94) had a history of prior abdominal surgeries, and over 63% of female patients had previously undergone hystero-annessiectomy.

### Intraoperative findings

**Figure 3:**
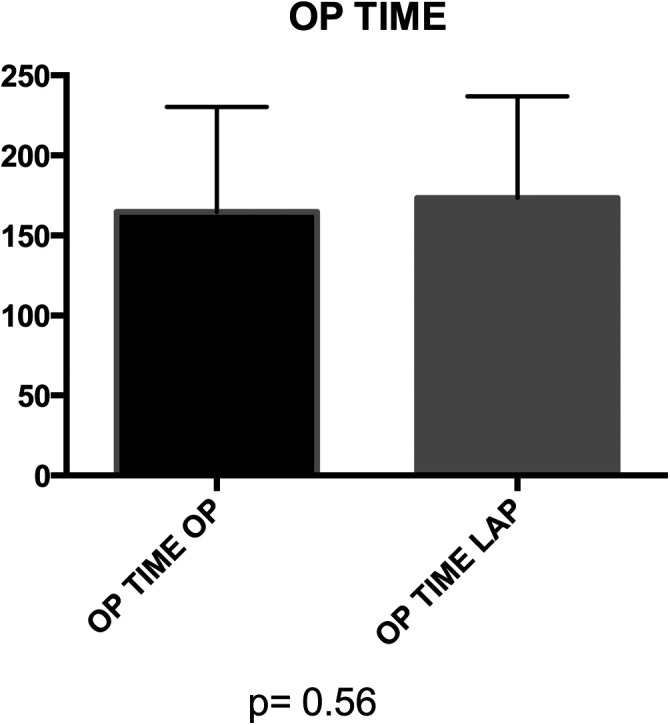
Group A (mean ± SD): [164.8 ± 11.22] group B (mean ± SD): [173.5 ± 9.8] *p*-value: [*p* = 0.56]. No statistically significant difference was found in the operation time distribution between Group A and Group B (*p* > 0.05).

**Figure 4:**
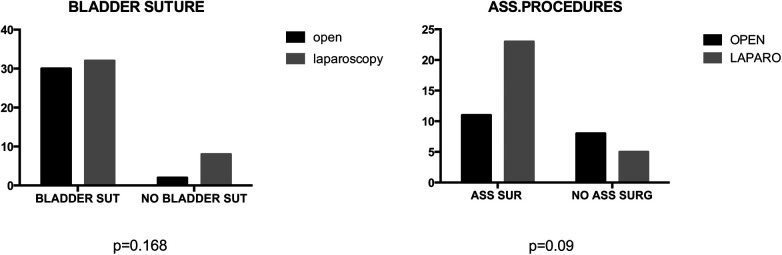
In the comparative analysis of two groups, comprising percentage of bladder suture or associated surgical procedures, no statistically significant differences were observed, with *p*-values exceeding 0.05.

**Figure 5:**
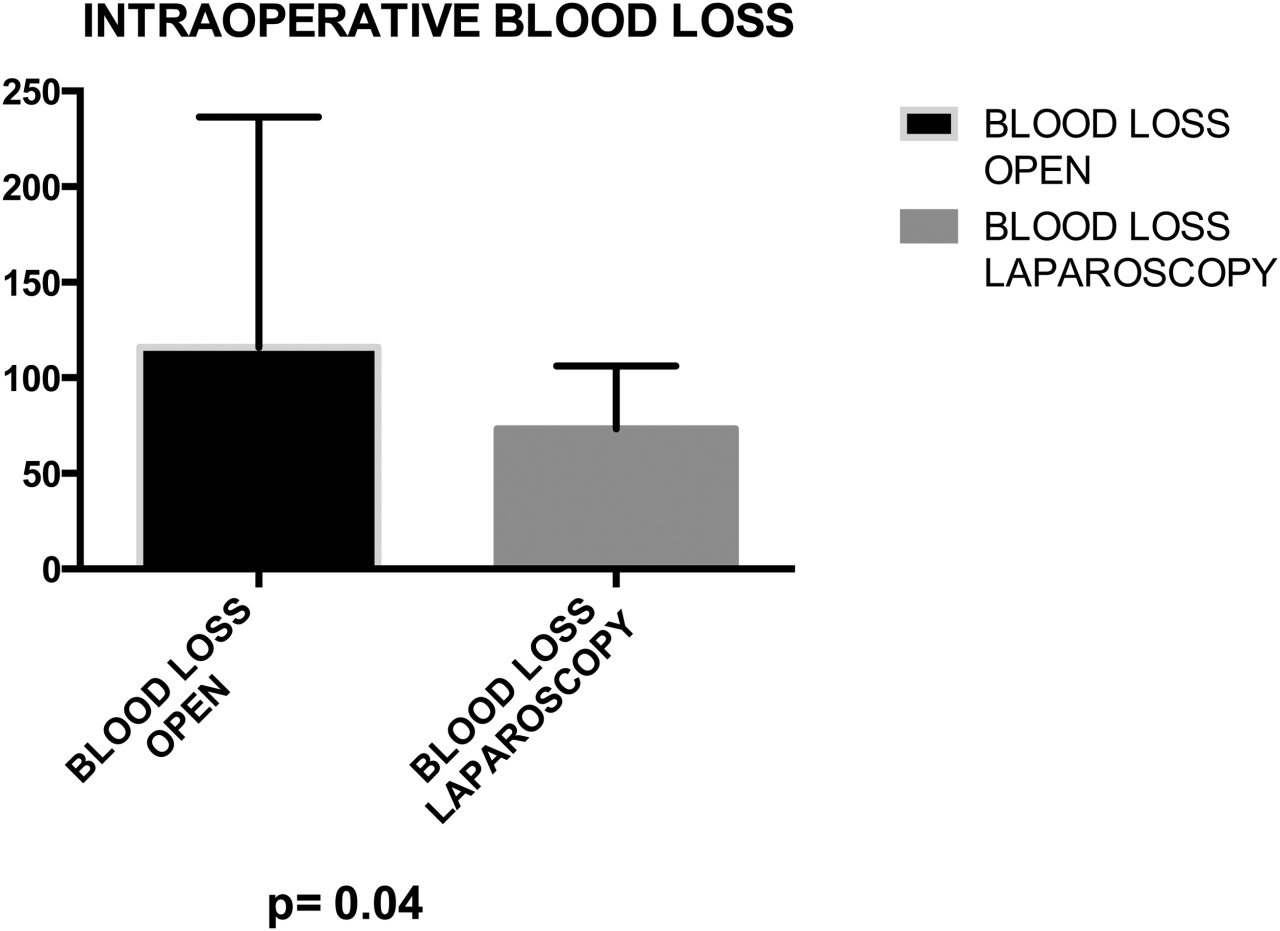
Group A (mean ± SD): [115.9 ± 20.35] group B (mean ± SD): [73.21 ± 5.08] *p*-value: [*p* = 0.04]. Group A exhibited a mean blood loss of 115.9 ± 20.35, while Group B showed 73.21 ± 5.08. The *p*-value of 0.04 suggests a statistically significant difference between the groups.

Mean operative time did not significantly differ between groups (164.8 vs. 173.7 min) (Figure 3). No statistical significance was observed between groups in terms of bladder suture and associated surgical procedures (Figure 4) Intraoperative blood loss was significantly higher in Group A (115.9 vs. 73.21 ml) (Figure 5). Sigmoid colectomy with IMA preservation was performed in 16 patients of Group B. Conversion to open surgery was required for 5% of Group B due to chronic tissue inflammation and severe fibrosis.

### Postoperative outcomes

**Figure 6:**
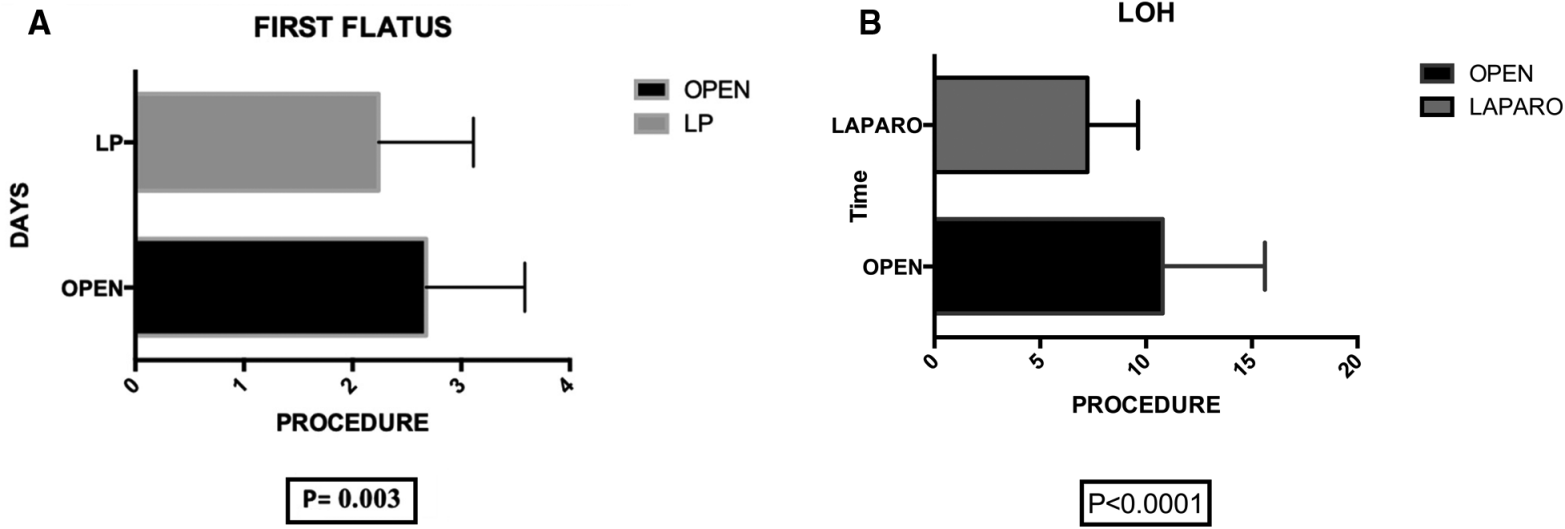
(**A**) Group A (mean ± SD): [2.67 ± 0.13] group B (mean ± SD): [2.23 ± 0.12] *p*-value: [0.003] the statistical analysis indicates a significant difference between group A (2.67 ± 0.13) and group B (2.23 ± 0.12) with a *p*-value of 0.003, suggesting a noteworthy variation in the measured parameter.” (**B**) group A (mean ± SD): [10.79 ± 0.88] group B (mean ± SD): [7.24 ± 0.36] *p*-value: [<0.0001]. The analysis reveals a significant difference in length of hospitalization (LOH) between group A (10.79 ± 0.88) and group B (7.24 ± 0.36) with a *p*-value of <0.0001, indicating a substantial disparity in the duration of hospital stay.

**Figure 7:**
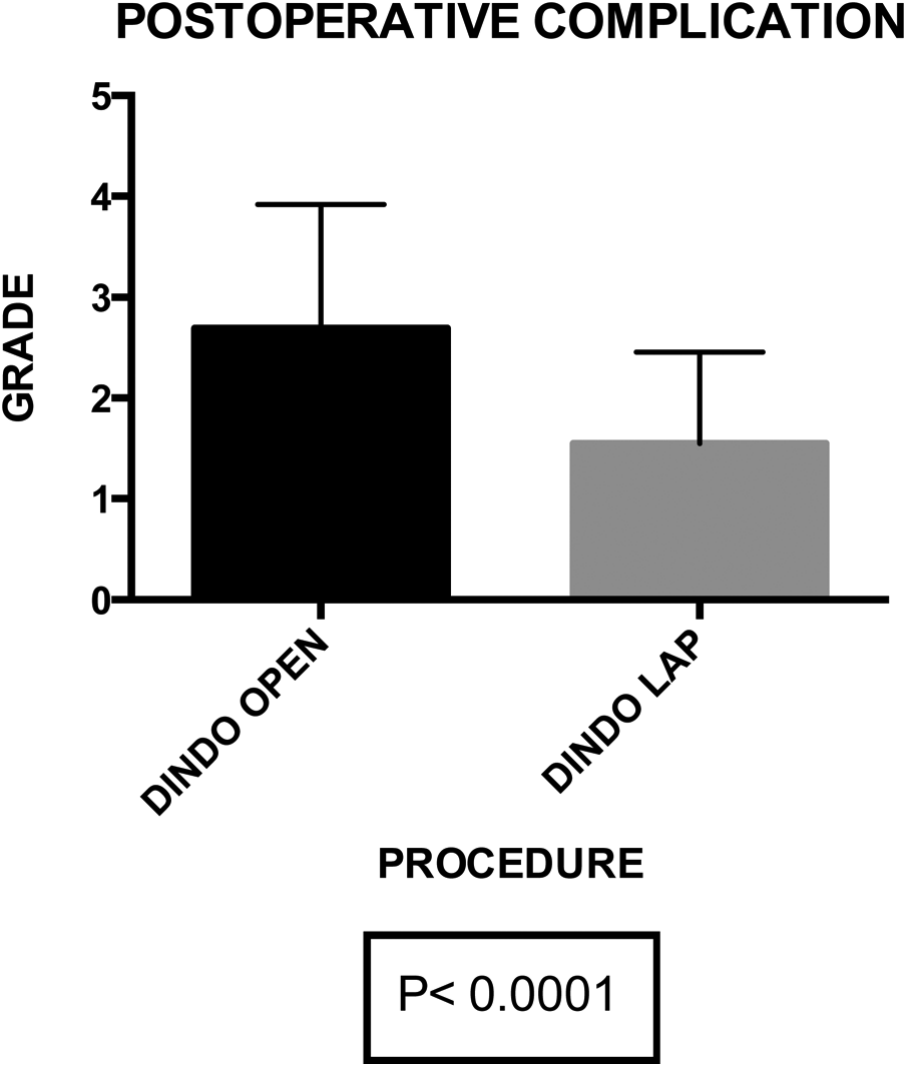
Group A (mean ± SD): [2.68 ± 0.21] group B (mean ± SD): [1.56 ± 0.14]. *p*-value: [*p* < 0.0001]. The post-operative complications exhibit a statistically significant difference between group A (2.68 ± 0.21) and group B (1.56 ± 0.14) with a *p*-value of <0.0001, highlighting a substantial variance in complication rates between the two groups.

**Figure 8:**
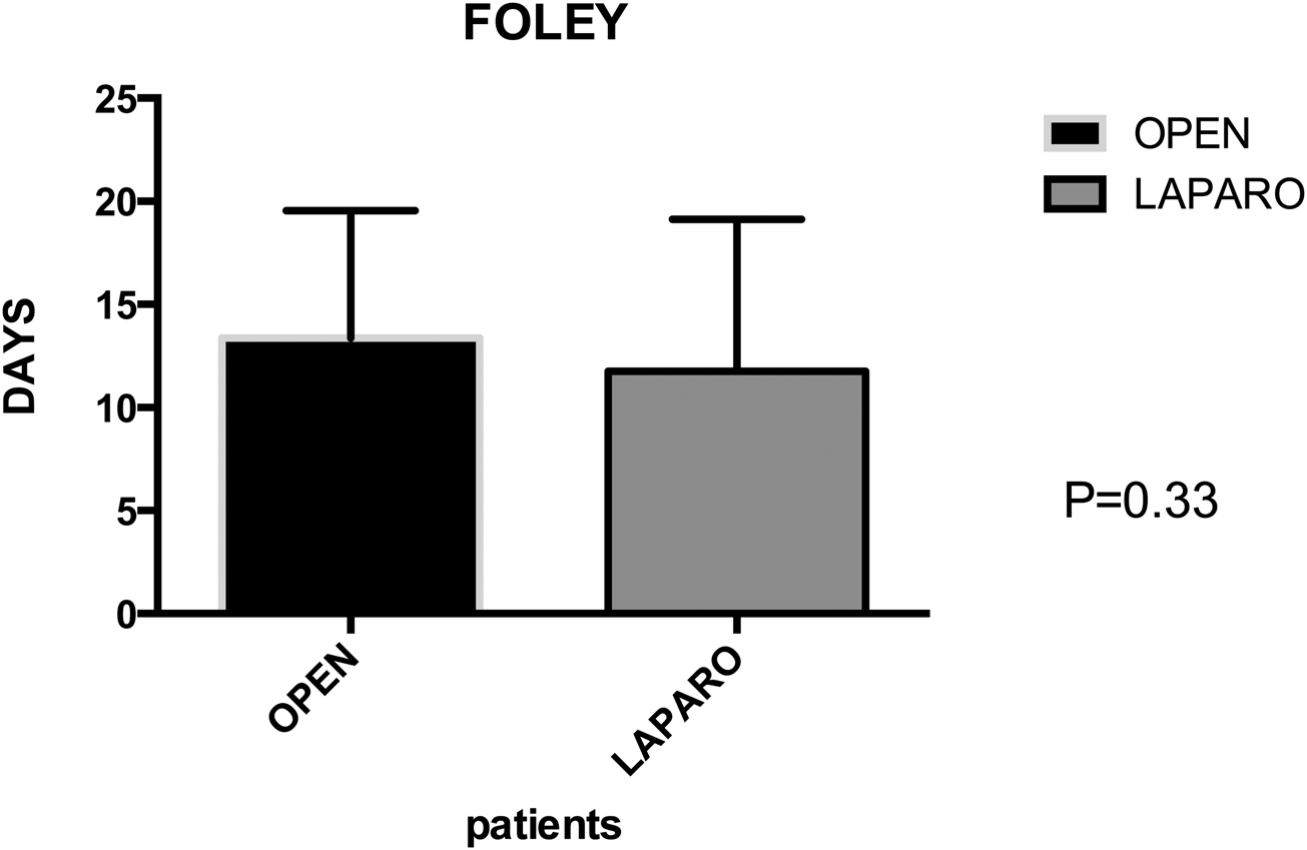
Group A (mean ± SD): [13.35 ± 1.12] group B (mean ± SD): [11.77 ± 1.12] *p*-value: [0.33 NS]. The permanence of urinary catheter demonstrates no statistically significant difference between group A (13.35 ± 1.12) and group B (11.77 ± 1.12), with a *p*-value of 0.33 (NS), indicating similarity in catheter duration between the two groups.

Postoperative primary ileus was significantly lower in the laparoscopic group (2.67 vs. 2.3 days; *P* = 0.003) (Figure 6). Overall postoperative morbidity (Clavien-Dindo classification grade 3 or higher) was 16.3%, with significantly higher morbidity in Group A (*P* < 0.0001) (Figure 7). No reoperation for postoperative complications was performed. Median time of Foley catheter removal was not statistically different between the two cohorts (13.35 vs. 11.77 days) (Figure 8). However, the median length of hospitalization was significantly shorter in patients who underwent laparoscopic procedures (7.2 vs. 10.7 days; *P* < 0.0001) (Figure 6). Mortality was observed in Group A (two patients), with an overall mortality of 2.6%. Notably, the two observed deaths were unrelated to surgical complications. After a two-year follow-up, no recurrence was observed.

## Discussion

In the realm of laparoscopic interventions for colovesical fistulas (CVF) complicating diverticular disease, our in-depth investigation emerges as a cornerstone, assuming even greater significance when situated within the broader landscape of existing research. The synthesis of insights from various studies, coupled with the recent systematic review by Cirocchi et al. (17), establishes a comprehensive foundation. Moreover, we incorporate crucial findings from three pivotal papers—by Badic et al. (11), N. L. Bertelson et al. (12) that not only enrich our understanding but also contribute essential perspectives to the ongoing discourse on this challenging condition.

Within our 76-patient cohort, evenly distributed between genders and categorized into Group A (undergoing open surgery) and Group B (undergoing laparoscopy), we robustly affirm the safety and efficacy of laparoscopy. Our emphasis on routine applicability in a homogeneous patient cohort underscores the potential versatility of the minimally invasive approach. Importantly, despite no significant differences in operative time, blood loss, and associated surgical procedures between the two groups, the laparoscopic approach showcases distinct advantages. These include reduced intraoperative blood loss (*P* = 0.04), diminished postoperative primary ileus (*P* = 0.003), lower postoperative morbidity (*P* < 0.0001), and a shorter median length of stay (*P* < 0.0001).

Crucially, our findings challenge historical perceptions regarding laparoscopy's time-intensive nature, with no significantly higher median operation times for laparoscopic resection compared to open surgery. The low conversion rate of 5%, notably lower than earlier studies, underscores the evolving landscape of laparoscopic techniques and the pivotal role of surgeon experience in mitigating conversion risks.

Cirocchi (17) and colleagues, through their systematic review, provide a panoramic view that likely encompasses diverse patient cohorts and procedural nuances across various studies. This collective evidence enriches our findings by offering nuanced insights into the evolving realm of laparoscopic interventions for CVF. Building upon this foundation, the insights presented by Badic et al. (11) in their paper, “Colovesical Fistula Complicating Diverticular Disease: A 14-Year Experience,” contribute a valuable 14-year experience in managing CVF. Their extensive retrospective analysis sheds light on long-term trends, challenges, and outcomes associated with laparoscopic interventions, thereby significantly broadening the temporal understanding of laparoscopic management for CVF.

Additionally, N. L. Bertelson et al.'s work in “Diverticular Colovesical Fistula: What Should We Really Be Doing?” (12) introduces a nuanced perspective on the current state of managing diverticular colovesical fistulas. By delving into the question of optimal practices, this paper addresses key considerations that not only inform but also complement our study. This valuable insight into the ongoing discourse on best practices enhances our understanding of how our findings align or diverge from current approaches. This multifaceted approach, incorporating insights from Cirocchi et al. (16), Bogdan Badic et al. (11), N. L. Bertelson et al. (12), contributes to a more comprehensive and nuanced understanding of laparoscopic interventions for CVF.

Delving into the systematic review by Cirocchi et al. (16), reveals a more nuanced exploration of parameters such as operative time, blood loss, conversion rates, and postoperative outcomes. By comparing our specific findings with this broader evidence base (18), a more comprehensive and nuanced understanding of laparoscopic benefits may emerge, thereby enhancing the applicability of these techniques in diverse clinical contexts.

The historical trajectory of laparoscopic resection for diverticular fistulizing disease, initiated in 1994 by Puente et al. (25). And subsequently reported by Hewett et al. (26) in 1995, underscores the initial challenges faced. Despite early promising experiences, the use of a mini-invasive technique for CVF caused by diverticular disease encountered obstacles due to the longer operative times of laparoscopy compared to open surgery and the high conversion rate (up to 60% in certain series) attributable to fibrosis and/or severe inflammation (16–32).

Our present study represents the largest series of laparoscopically treated CVF to date, distinguishing itself by exclusively including patients affected by CVF secondary to diverticular disease. Notably, there are few reports in the literature focusing exclusively on CVF by diverticulitis (33, 34), with most published studies encompassing fistulas of mixed etiology (35) or mixed diverticular fistula (16).

Furthermore, the inclusion of patients who were not previously selected, with a significant percentage (57.8%) having undergone previous abdominal operations, adds a real-world dimension to our study. The recent review by Keady and co-worker (10) on morbidity and mortality in the surgical management of CVF reported variable rates across studies. In our series, we observed an overall morbidity of 14.9%, significantly higher in the open surgery group. Remarkably, mortality was zero in the laparoscopic group, attributed to the high volume of laparoscopic operations performed and the adherence to standardized procedures by the same surgeons.

In contrast to previous reports (10, 11–34), the median operation time for laparoscopic resection for CVF was not significantly higher than the time for open surgery. Notably, the low conversion rate of 5% in the present cohort, considerably lower than rates reported in previous studies (10–16), reflects the evolving experience of surgeons and advancements in surgical techniques. Conversion was necessary in two patients due to severe fibrosis, impeding safe dissection. One of these patients presented with a concomitant colovaginal fistula, highlighting the complexity of cases. Engledow et al. (36) reported different rates of conversion over a period of 10 years (64% vs. 29%) based on surgeon experience. Accordingly, Kockerling et al. (37) concluded that laparoscopy, for fistulizing diverticular disease, should only be carried out by experienced laparoscopic surgeons.

The advantages of the laparoscopic approach in terms of intraoperative blood loss (*P* = 0.04), postoperative primary ileus (*P* = 0.003), and median hospitalization (*P* < 0.0001) further underscore its potential to offer the benefits of minimally invasive surgery to patients with CVF due to diverticular disease. This reaffirms and extends the findings proposed in previous studies (10–17).

In summary, our study, interwoven with insights from pivotal research and guided by a robust patient cohort, contributes significantly to the evolving discourse on laparoscopic interventions for CVF complicating diverticular disease. Through a comprehensive exploration of historical challenges, contemporary advantages, and nuanced comparisons, our findings provide valuable considerations for future research and clinical practice in the management of this challenging condition.

### Study limitations

While our study contributes valuable insights into the laparoscopic management of colovesical fistulas (CVFs) secondary to diverticular disease, it is essential to acknowledge certain limitations that temper the generalizability and depth of our findings. The retrospective nature of our study introduces inherent limitations. Reliance on historical data and medical records may result in incomplete or biased information, potentially impacting the accuracy of our conclusions.

Our study draws exclusively from the experience of two institutions, potentially limiting the external validity of our findings. Variations in patient demographics, surgical practices, and institutional protocols may not fully capture the diversity encountered in broader healthcare settings.

While our study cohort provides valuable insights, the relatively modest sample size may constrain the robustness of our conclusions. Larger-scale, multi-center studies would offer a more comprehensive perspective on the nuances of laparoscopic interventions for CVFs.

Over the decade covered by our study, surgical techniques and practices may have evolved. Technological advancements and changes in clinical approaches could impact the relevance of our early data. While emphasizing the importance of surgeon experience, our study does not delve deeply into the specifics of individual experience levels. Variability in surgeon experience may contribute to outcome disparities that are not fully explored in our analysis.

External factors, such as advancements in perioperative care, shifts in patient demographics, or changes in healthcare policies, are not comprehensively considered. These external dynamics, beyond the scope of our study, could influence outcomes.

Our study focuses primarily on the comparison between laparoscopic and open surgery, neglecting exploration of other emerging modalities or technologies in the field of minimally invasive interventions.

Certain patient-related variables, including comorbidities, socioeconomic factors, and patient preferences, remain largely unexplored in our analysis. These variables may play a significant role in treatment choices and outcomes.

In acknowledging these limitations, we aim to provide a transparent context for the interpretation of our study findings, encouraging future research to address these constraints for a more comprehensive understanding of laparoscopic interventions for CVFs.

## Conclusions

In the crucible of limitations, our study emerges unyielding, a force challenging the status quo in laparoscopic interventions for colovesical fistulas complicating diverticular disease. Amidst the retrospective constraints, our findings act as a catalyst for change, daring the medical community to break free from tradition. We beckon towards a future where innovation trumps limitations, urging a paradigm shift in the approach to colovesical fistula treatment.

## Data Availability

The raw data supporting the conclusions of this article will be made available by the authors, without undue reservation.
